# Epigenetic intersection of BDNF Val66Met genotype with premenstrual dysphoric disorder transcriptome in a cross-species model of estradiol add-back

**DOI:** 10.1038/s41380-018-0274-3

**Published:** 2018-10-24

**Authors:** Jordan Marrocco, Nathan R. Einhorn, Gordon H. Petty, Howard Li, Neelima Dubey, Jessica Hoffman, Karen F. Berman, David Goldman, Francis S. Lee, Peter J. Schmidt, Bruce S. McEwen

**Affiliations:** 1 Laboratory of Neuroendocrinology, The Rockefeller University, New York, NY USA; 2 Behavioral Endocrinology Branch, National Institute of Mental Health, Bethesda, MD USA; 3 Dr. D. Y. Patil Biotechnology & Bioinformatics Institute, Pune, India; 4 Uniformed Services University of the Health Sciences, Bethesda, MD USA; 5 Section on Integrative Neuroimaging, National Institute of Mental Health, Bethesda, MD USA; 6 Laboratory of Neurogenetics, National Institute on Alcohol Abuse and Alcoholism, Bethesda, MD USA; 7 Department of Psychiatry, Sackler Institute for Developmental Psychobiology, Weill Cornell Medical College, New York, NY USA

**Keywords:** Predictive markers, Neuroscience

## Abstract

Premenstrual dysphoric disorder (PMDD) affects over 5% of women, with symptoms similar to anxiety and major depression, and is associated with differential sensitivity to circulating ovarian hormones. Little is known about the genetic and epigenetic factors that increase the risk to develop PMDD. We report that 17β-estradiol (E2) affects the behavior and the epigenome in a mouse model carrying a single-nucleotide polymorphism of the brain-derived neurotrophic factor gene (BDNF Val66Met), in a way that recapitulates the hallmarks of PMDD. Ovariectomized mice heterozygous for the BDNF Met allele (Het-Met) and their matched wild-type (WT) mice were administered estradiol or vehicle in drinking water for 6 weeks. Using the open field and the splash test, we show that E2 add-back induces anxiety-like and depression-like behavior in Het-Met mice, but not in WT mice. RNA-seq of the ventral hippocampus (vHpc) highlights that E2-dependent gene expression is markedly different between WT mice and Het-Met mice. Through a comparative whole-genome RNA-seq analysis between mouse vHpc and lymphoblastoid cell line cultures from control women and women with PMDD, we discovered common epigenetic biomarkers that transcend species and cell types. Those genes include epigenetic modifiers of the ESC/E(Z) complex, an effector of response to ovarian steroids. Although the BDNF Met genotype intersects the behavioral and transcriptional traits of women with PMDD, we suggest that these similarities speak to the epigenetic factors by which ovarian steroids produce negative behavioral effects.

## Introduction

Sex hormones and their neurosteroid metabolites contribute to the establishment of sex differences as well as the regulation of mental state [1, 2]. In most women, natural fluctuations in circulating estrogens across the menstrual cycle are associated with mood and cognitive changes. Of note, 3–8% of menstruating women meet diagnostic criteria for premenstrual dysphoric disorder (PMDD), a psychiatric condition that consists of a cluster of affective, behavioral, and somatic symptoms that recur monthly during the luteal phase of the menstrual cycle [3, 4]. The individual variability in the impact of ovarian hormones on brain and behavior depends on several contextual factors including age, environmental influences (e.g., exposure to stress), and genetic risk.

Variation in the gene coding for brain-derived neurotrophic factor (BDNF) has been associated with estrogen-dependent hippocampal function, anxiety, and sex differences both in human and experimental models [5–8]. BDNF mediates the effects of estradiol (E2) in the hippocampus [9]. In female mice and rats, the E2 surge during the estrous cycle induces BDNF mRNA and protein, and increases the activation of the BDNF tyrosine kinase receptor, TrkB, in the hippocampus [10–13]. Interestingly, TrkB and steroid hormone receptors co-localize in the hippocampus and prefrontal cortex, suggesting converging functional actions of steroid hormones and BDNF in the brain [14]. Mice with a single-nucleotide polymorphism (SNP) of the human *BDNF* gene (Val66Met; rs6265), which results in an amino acid change from a valine to a methionine at position 66, show a negative behavioral traits to ovarian hormone fluctuations [5, 7], resembling that of women with PMDD to estrogens.

To investigate this parallel, we tested the behavioral effects of E2 and the BDNF Met genotype in ovariectomized (OVX) mice treated with E2 in drinking water to minimize the stress of the injection. To relate the behavioral results to change in gene expression, we examined a whole-genome transcriptome analysis of the ventral hippocampus (vHpc). For translational purposes, we also conducted a comparative whole-genome RNA-seq analysis between mouse vHpc and lymphoblastoid cell line (LCL) cultures from healthy women and women with PMDD, focusing on shared epigenetic biomarkers that transcend species and cell types and may indicate vulnerability for PMDD.

## Materials and methods

### Animals

BDNF Val66Met knock-in mice were generated in the Lee laboratory, as previously described [15]. Wild-type C57/BL6 mice (WT) and heterozygous BDNF Val66Met knock-in mice (Het-Met) were obtained by performing in-house breeding. Female mice around 70 to 80-days-old were ovariectomized (see Supplementary Materials) and randomly assigned to vehicle or E2 add-back groups. After 2 weeks of treatment, mice were tested for anxiety-like and depression-like behavior using the open field test and splash test, respectively (see Supplementary Materials).

### Humans

Women with PMDD, control women, and their respective lymphoblastoid cell lines (LCLs) were included in this study based on the criteria of Schmidt et al. [16] (see Supplementary Materials). EBV-transformed LCLs were made from peripheral blood as previously described [17], and cultured under identical conditions [18]. The distribution of the BDNF genotype amongst subjects is specified in the Supplementary Material and Methods.

### qRT-PCR

Mice were killed by cervical dislocation and rapidly decapitated to extract the hippocampi. The uteri were removed and weighed to assess the efficacy of E2 add-back. The vHpc was isolated as described in Marrocco et al. [19]. RNA was extracted from ventral hippocampal tissue using the Qiagen Lipid Tissue Mini Kit (ThermoFisher, Waltham, MA, USA). RNA was transcribed to cDNA using High Capacity cDNA Reverse Transcription Kit (Applied Biosystems, Warrington, UK). cDNA was used to perform qRT-PCRs to measure the expression levels of *Aebp2*, *Eed*, *Ezh1*, *Ezh2*, *Hdac2*, *Jarid2*, *Mtf2*, *Phf1*, *Phf19*, *Rbbp4*, *Rbbp7*, *Sirt1*, and *Suz12*. Taqman Universal PCR master mix (Applied Biosystems, Warrington, UK) was used with all primers and run on a Quantstudio 12k flex thermocycler (Life Technologies, Carlsbad, CA, USA). Fold change was calculated using the 2^-ΔΔCT^ method and normalized to *Sdha* levels.

### RNA-sequencing

#### Mouse

A total of 200 ng of RNA per group was prepared for sequencing by The Rockefeller University Genomics Core Facility using the Tru-Seq Stranded RNA-Seq library preparation kit (Illumina, USA). Three biological replicates were used per experimental group, comprising of RNA pooled from two animals each. cDNA libraries were sequenced on an Illumina NextSeq 500 in a single lane to obtain 75-bp single-end reads at an approximate sequencing depth of 35–40 million reads per sample.

#### Human

RNA-seq was performed in hormone-treated and untreated (steroid-free) LCLs from women with PMDD and Controls. Hormone-treated LCLs were exposed for 24 h in vitro to 100 nmol E2. cDNA libraries were sequenced on an Illumina Genome Analyzer (GAIIx) for LCLs at a sequencing depth of 17.7 million reads per sample at 36-bp [18].

### Sequencing analysis and statistics

Raw reads were trimmed, filtered, and aligned to mouse genome (mm10) using TopHat2 [6]. Differential expression analysis (average of three samples per group) was conducted with Strand NGS software (Agilent Technologies, USA), in which DESeq was used to quantify transcript reads and obtain *z*-scores and fold change values for individual genes. Genes with *p* < 0.05, Benjamini–Hochberg false discovery rate corrected, and fold change <1.3 were selected for further analysis. Differences in integrated read density were visualized against the mouse genome by using the Venn diagram and heat map tools of Strand NGS. GO categories were manually curated from results of the Database for Annotation, Visualization and Integrated Discovery (DAVID) functional annotation cluster tool. Microsoft Excel (Microsoft, USA) was used to obtain gene expression profiles by sorting genes based on fold change. To compare the human and mouse genomes, a DESeq2 analysis pipeline through R was used to quantify both species’ raw reads and obtain *p*-values and log fold changes. Using Microsoft Excel, human genes were matched to their mouse orthologues and filtered with an uncorrected *p* < 0.05. Behavioral data were analyzed using GraphPad Prism (GraphPad Software, Inc., USA) by performing a two-way ANOVA followed by Neumann–Keuls post hoc analysis. A *p*-value < 0.05 was considered as statistically significant.

## Results

### E2 add-back induces anxiety-like and depressive-like behavior in OVX Het-Met mice

**Fig. 1:**
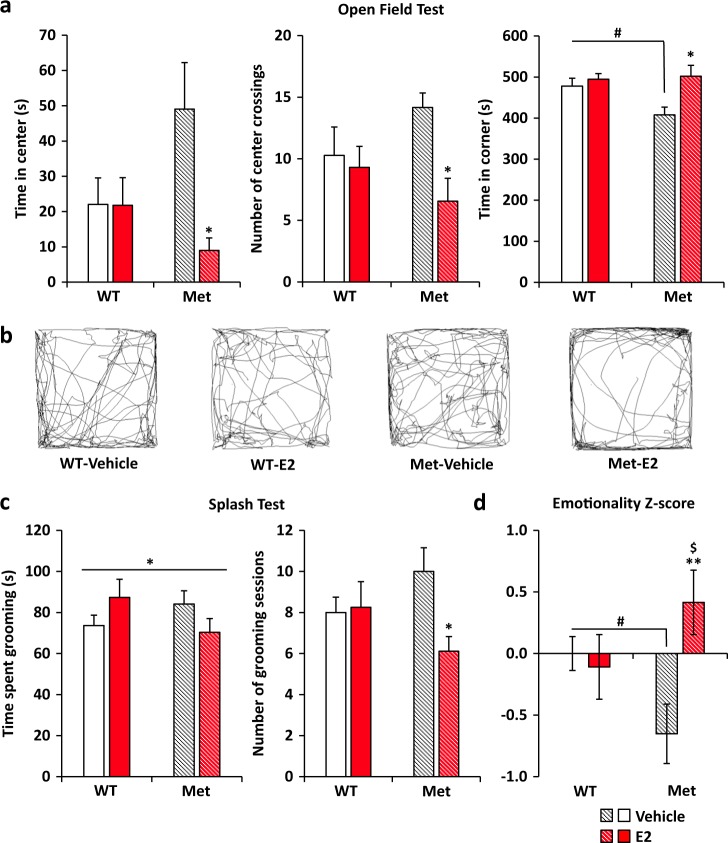
Behavioral impairment in OVX BDNF Val66Met mice induced by E2 add-back. **a** Open-field test shows increased anxiety-like behavior in OVX Het-Met mice after E2 add-back when compared to OVX WT mice. (2-way ANOVA, time in the center: genotype-by-treatment: F_(1,38)_ = 4.39, *p* < 0.05; number of center crossings: treatment: F_(1,38)_ = 5.66, *p* < 0.05; treatment: time in corner: F_(1,38)_ = 7.75, *p* < 0.01). **b** Representative tracking of the path of one mouse per group in the open field test. **c** Splash test showed that OVX Het-Met mice treated with E2 exhibited depressive-like behavior. (2-way ANOVA, genotype-by-treatment: time spent grooming: F_(1,35)_ = 4.23, *p* < 0.05; genotype-by-treatment: number of grooming sessions: F_(1,35)_ = 4.49, *p* < 0.05). **d** OVX Het-Met mice treated with vehicle displayed lower emotionality score than OVX WT mice. E2 treatment increased emotionality selectively in OVX Het-Met mice. (2-way ANOVA, genotype-by-treatment: F_(1,41)_ = 10.03, *p* < 0.01). Columns represent the mean ± S.E.M. of 9–12 determinations per group. *p < 0.05, **p < 0.01, ***p < 0.001 vs matched vehicle-treated mice. ^#^*p* < 0.05 vs vehicle-treated WT mice. ^$^*p* = 0.06 vs E2-treated WT mice. WT OVX WT mice, Met OVX Het-Met mice

At day 14 of treatment (i.e., E2 or vehicle), anxiety-like behavior was assessed using the light-dark box and open field tests. Mice displayed no differences in the time spent in the light box or the latency to enter the light box (Supplementary Table 1). We then used the open field test and recorded the amount of time each mouse spent in the center of the arena versus the time spent in the corners. This test has validity in showing genotype differences in anxiety-like behavior in Met carriers [5, 7]. E2-treated OVX Het-Met mice spent less time in the center and more time in the corners when compared to vehicle-treated OVX Het-Met mice. The number of center crossings was also lower in E2-treated OVX Het-Met mice when compared to vehicle-treated OVX Het-Met mice. Interestingly, vehicle-treated OVX Het-Met mice spent significantly less time in the corners and showed a tendency to spend more time in the center of the arena when compared to OVX WT mice, regardless of treatment (Fig. 1a, b). Thus, E2 add-back induced anxiety-like behavior in OVX Het-Met mice but not in OVX WT mice.

Depression-like behavior was measured by recording the number of grooming sessions and the time spent grooming in the splash test, where reduced grooming signifies increased depressive-like behavior [20, 21]. E2 treatment increased the time spent grooming in OVX WT mice, whereas it decreased in OVX Het-Met mice. Also, the number of grooming sessions were significantly less in E2-treated OVX Het-Met mice when compared to vehicle-treated OVX Het-Met mice, indicating that E2 add-back induced depressive-like behavior only in Met carriers (Fig. 1c).

To increase the sensitivity and reliability of our measures for the behavioral phenotype, we applied z-normalization across complementary measures of emotionality, i.e., anxiety-like and depression-like behaviors [22]. Interestingly, the distribution of individual *z*-score revealed a pattern of decreased emotionality in vehicle-treated OVX Het-Met mice when compared to vehicle-treated OVX WT mice. E2-treated Het-Met mice showed higher emotionality when compared to E2-treated WT mice. In addition, E2 treatment increased the *z*-score selectively in OVX Het-Met mice, corroborating the finding that E2 affects emotional behavior in a genotype-specific manner (Fig. 1d).

### Het-Met mice have a unique transcriptional profile and distinct response to E2 treatment in the vHpc

**Fig. 2:**
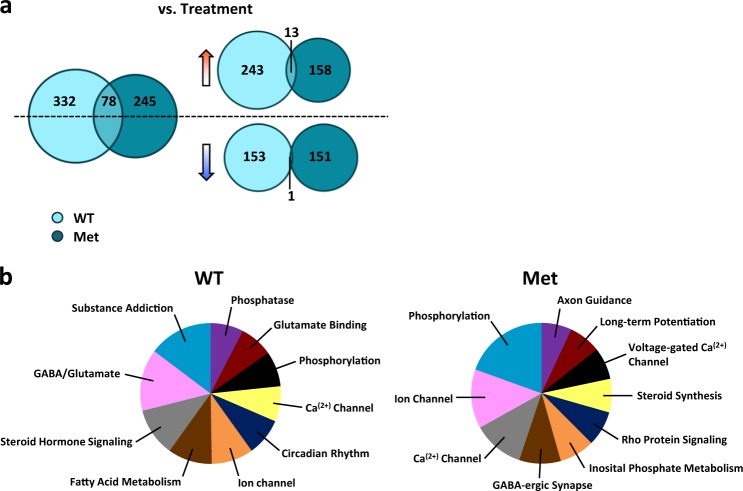
Hippocampal whole-genome sequencing in E2-treated OVX mice. **a** Venn diagram depicting the number of genes altered in E2-treated OVX WT mice (light blue), E2-treated OVX Het-Met mice (dark blue circle), and in both treatments (blue overlap) (*Z*-score < 0.05; absolute fold change > 1.3) when compared with genotype-matched OVX mice. E2-induced distinct gene expression changes in OVX WT mice and OVX Het-Met mice. We found that E2 induced 332 genes in WT mice and 245 genes in Het-Met mice, with 78 E2-induced genes common to both genotypes. Separating these E2-regulated genes based on the direction of their fold change revealed that E2 upregulated 243 genes in WT mice and 158 genes in Het-Met mice, with only 13 genes commonly upregulated in both genotypes. In addition, 153 genes were downregulated in WT mice and 151 downregulated in Het-Met mice by E2, with only 1 gene, *Myoc*, common to both genotypes (See Supplementary Table 2). *Myoc* was used to validate the RNA-seq bioinformatics prediction since it was common across all comparisons and among the top 100 most differentially expressed genes (Supplementary Fig. 3). **b** Pie-charts depicting the enrichment analysis of the top 10 pathways from Database for Annotation, Visualization and Integrated Discovery (DAVID) revealed several gene pathways related to cell signaling, steroid hormones, and neuronal function, such as circadian rhythm, glutamate-GABA system, long-term potentiation, axon guidance, and synapse. Pathways were obtained by analyzing the genes in (clockwise): light blue circle and dark blue circle in **a**

RNA-seq of the vHpc, which encodes memory associated with the stress response and mood disorders [23–25], revealed that E2 add-back induced only 78 genes common to OVX WT and OVX Het-Met mice, and even less so when filtering by fold change directionality, indicating that E2 regulation produces unique gene expression patterns in WT and in Het-Met mice (Fig. 2a; Supplementary Table 2). To further investigate the differential effect of genotypes, we compared OVX WT and OVX Het-Met mice within treatments. This comparison highlighted that there exists an intrinsic, distinct hippocampal gene expression in each genotype regardless of circulating endogenous E2 (Supplementary Fig. 2A; Supplementary Table 3).

Using Database for Annotation, Visualization and Integrated Discovery (DAVID) functional annotation cluster tool, differentially expressed genes were clustered based on gene ontology. E2 induced markedly different gene networks between OVX WT and OVX Het-Met mice. For example, E2-treated OVX WT mice displayed enrichment in pathways that were not present in E2-treated OVX Het-Met mice, such as circadian rhythm and fatty acid metabolism (Fig. 2b).

**Fig. 3:**
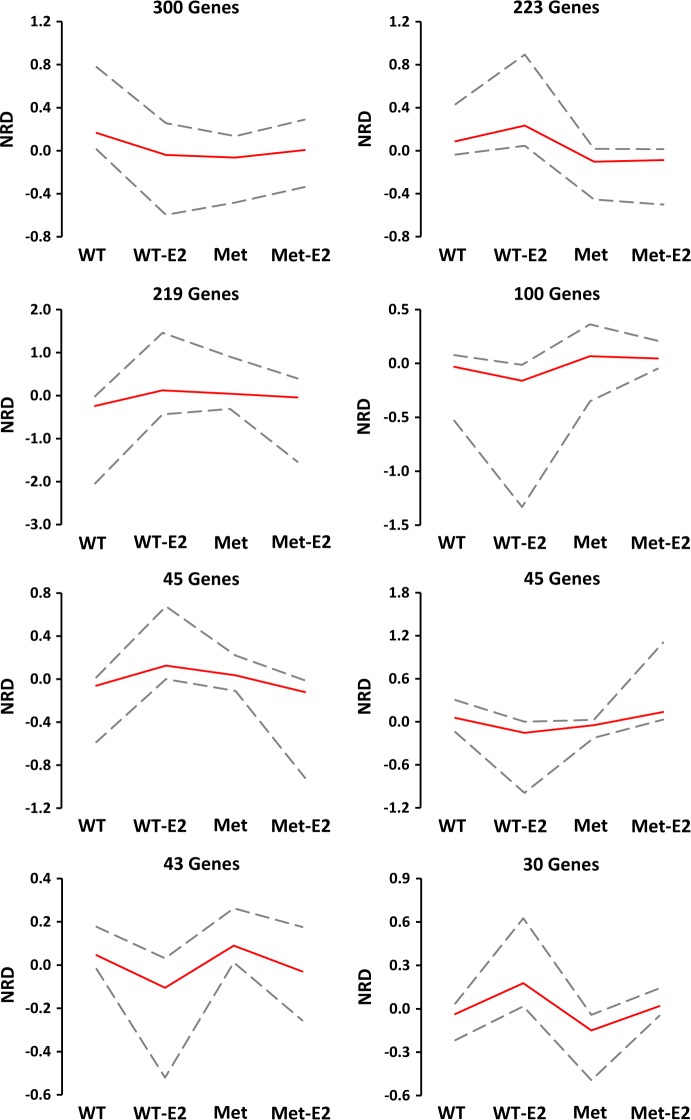
Clustering of genes by expression profiles. Genes were clustered based on their fold change in OVX WT-E2, Het-Met, and Het-Met-E2 versus WT. Significance was determined by using a *p*-value < 0.05. Genes with the same expression profile were clustered together. The maximum, mean, and minimum normalized read density for each clustered profile is presented as a line graph. WT: OVX WT mice treated with vehicle. Met: OVX Het-Met mice treated with vehicle. WT-E2: OVX WT mice treated with E2. Met-E2: OVX Het-Met mice treated with E2

We then assessed gene expression patterns across treatment and genotypes. Genes were thresholded based on the direction of their fold change (*p*-value < 0.05) in E2-treated OVX WT, vehicle-treated OVX Het-Met, and E2-treated OVX Het-Met mice versus vehicle-treated OVX WT mice. Genes with the same expression profile were clustered together, and eight distinct expression patterns were found. Over 300 genes were downregulated by E2 in OVX WT mice, and upregulated or unchanged by E2 in OVX Het-Met mice. Another cluster of 223 genes was upregulated by E2 in OVX WT mice and unchanged by E2 in OVX Het-Met mice. Within these profiles, i.e., altered by E2 in OVX WT mice but unchanged by E2 in OVX Het-Met mice, we found the immediate-early genes of the *Egr* family *Egr3* and *Egr4*, the epigenetic modifiers *Nat8l, Setd7, Mecp2*, and *Phf10*, and the glutamatergic receptor *Gria2*. Curiously, the majority of genes were expressed similarly in OVX WT treated with E2 and vehicle-treated OVX Het-Met mice. This was reminiscent of the “pre-stressed state” of Het-Met mice where the same genes that are expressed without applied stress are also induced in WT mice by an acute stressor [6]. Only a minority of genes (a cluster of 43 genes and a cluster of 30 genes) was regulated similarly by E2 in both OVX WT and OVX Het-Met mice (Fig. 3), indicating that there was a small E2 effect independent of genotype. Conversely, we observed a much larger genotype-specific response to E2, as demonstrated by the several unique and distinct gene networks in the vHpc of Met carriers compared to WT mice.

### Epigenetic regulation in response to E2 add-back

**Fig. 4:**
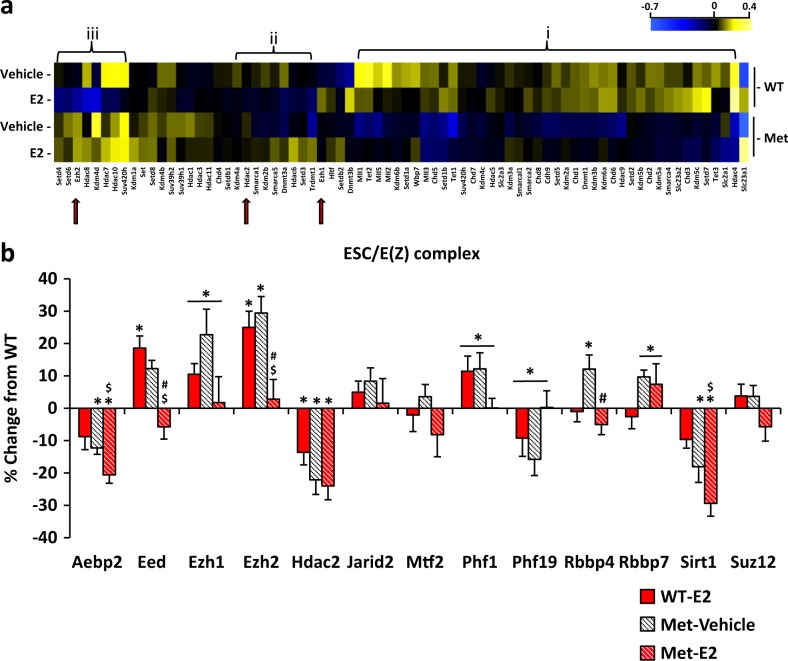
E2 effects on epigenetic genes and ESC/E(Z) complex. **a** Heat map represents the normalized read densities of 74 epigenetic genes, including 3 genes of the ESC/E(Z) complex (red arrows). Genes were clustered based on similar expression profiles: (i) Genes downregulated in OVX Het-Met mice when compared to OVX WT mice; (ii) and (iii) Genes downregulated by E2 in OVX WT mice and upregulated or unchanged by E2 in OVX Het-Met mice. **b** Percent change from OVX WT mice of mRNA expression measured by qRT-PCR for the 13 genes of the ESC/E(Z) complex. *Aebp2* was downregulated in OVX Het-Met mice regardless of treatment when compared to OVX WT mice. E2 had opposite genotype-by-treatment effects on the levels of *Eed, Ezh1/2, Phf1*, and *Phf19*. *Eed, Ezh1/2*, and *Phf1* were upregulated in E2-treated OVX WT mice, and downregulated in E2-treated OVX Het-Met mice, whereas *Phf19* was downregulated in E2-treated OVX WT mice, and upregulated in E2-treated OVX Het-Met mice. The histone deacetylase gene *Hdac2* was downregulated in E2-treated OVX WT mice but E2 had no effect on the levels of *Hdac2* in OVX Het-Met mice. The genes encoding for CAF1 histone-binding proteins RBBP4 and RBBP7 were both upregulated in vehicle-treated OVX Het-Met mice when compared to OVX WT mice. Of note, E2-induced downregulation of *Rbbp4* in OVX Het-Met mice, but had no effects on the levels of *Rbbp7*. Furthermore, the NAD-dependent deacetylase sirtuin-1 gene, *Sirt1*, was selectively downregulated in OVX Het-Met mice regardless of treatment. No directional changes were observed in *Jarid2*, *Mtf2*, and *Suz12*. Two-way ANOVA followed by Newman–Keuls post hoc was used to establish statistical significance for *Aebp2* (treatment: F_(1,25)_ = 8.17, *p* < 0.01; genotype: F_(1,25)_ = 16.27, *p* < 0.01), *Eed* (genotype-by-treatment: F_(1,25)_ = 19.24, *p* < 0.01), *Ezh1* (genotype-by-treatment: F_(1,24)_ = 6.80, *p* < 0.05), *Ezh2* (genotype-by-treatment: F_(1,21)_ = 23.53, *p* < 0.01), *Hdac2* (genotype: F_(1,25)_ = 16.03, *p* < 0.01), *Phf1* (genotype-by-treatment: F_(1,26)_ = 8.22, *p* < 0.01), *Phf19* (genotype-by-treatment: F_(1,21)_ = 5.51, *p* < 0.05), *Rbbp4* (genotype-by-treatment: F_(1,25)_ = 6.14, *p* < 0.05; treatment: F_(1,25)_ = 7.84, *p* < 0.01), *Rbbp7* (genotype: F_(1,23)_ = 4.68, *p* < 0.05), and *Sirt1* (treatment: F_(1,25)_ = 6.99, *p* < 0.05; genotype: F_(1,25)_ = 22.84, *p* < 0.01). Asterisk indicates vs vehicle-treated WT mice; dollar indicates E2-treated Het-Met mice vs E2-treated WT mice; hash indicates E2-treated Het-Met mice vs vehicle-treated Het-Met mice. Met-Vehicle: OVX Het-Met mice treated with vehicle. WT-E2: OVX WT mice treated with E2. Met-E2: OVX Het-Met mice treated with E2

We then assessed whether differential gene expression change in the Met carriers and in response to E2 add-back was associated with differentially expressed epigenetic modifiers in order to assess regulatory factors that are likely to be operative in all cells. Seventy-four epigenetic genes including ATP-dependent chromatin remodelers, chromatin helicase DNA binding proteins, DNA methylation and demethylation, histone deacetylases, histone demethylases, and histone methyltransferases genes [26] were clustered into a heat map cladogram. Expression of one cluster of epigenetic modifiers was lower in OVX Het-Met mice regardless of treatment when compared to OVX WT mice. In another cluster, E2 had an opposite effect in OVX Het-Met and OVX WT mice, i.e., genes were upregulated in E2-treated OVX Het-Met mice and downregulated in E2-treated OVX WT mice. A third cluster included genes that were downregulated by E2 add-back in OVX WT mice and unaltered by E2 add-back in OVX Het-Met mice (Fig. 4a). Thus, OVX WT and OVX Het-Met mice displayed distinct epigenetic signatures at baseline and in response to E2 add-back.

Importantly, epigenetic modifiers are implicated in ovarian hormone-related disorders [18, 27]. For example, Extra Sex Combs/Enhancer of Zeste (ESC/E(Z)) complex is a gene silencing network that methylates lysine-27 and lysine-9 residues of histone H3 to regulate transcription. This epigenetic complex consists of four core proteins (EZH2, SUZ12, EED, and RBBP4) and nine additional proteins (AEBP2, EZH1, HDAC2, JARID2, MTF2, PHF1, PHF19, RBBP7, and SIRT1) that orchestrate the entire network [28].

Based on the RNA-seq bioinformatic epigenetic predictions, that included some of the ESC/E(Z) complex genes, we used RT-qPCR on a replication of E2-add back. Results, summarized in Fig. 4, showed differential ESC/E(Z) complex regulation in the two genotypes. Since epigenetic modifiers are expressed across cell types and species, we next looked at the regulation of these genes in human cells.

### Het-Met mice intersect the epigenetic transcriptome of women with PMDD

**Fig. 5:**
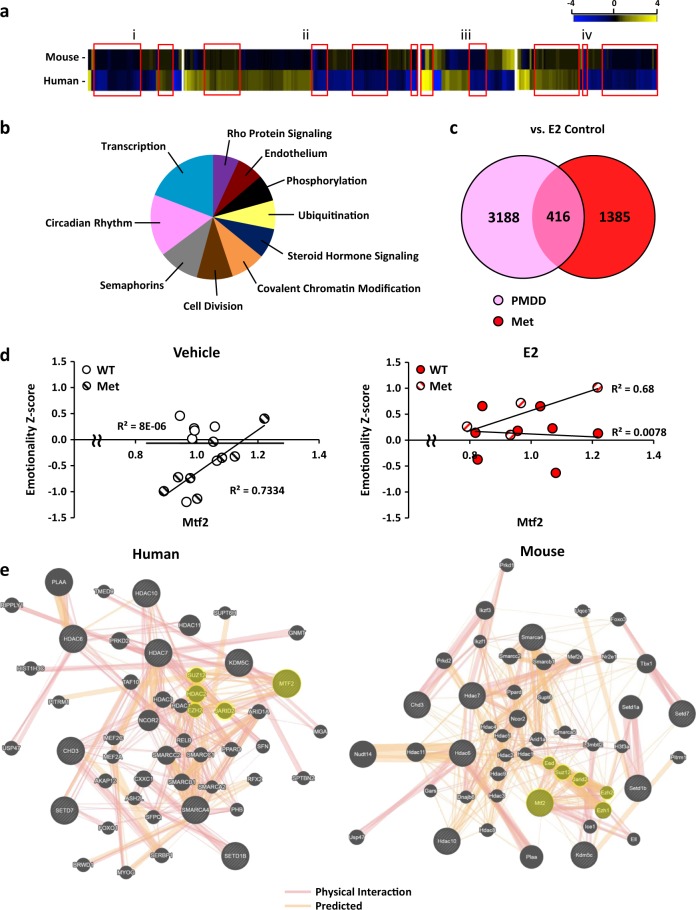
Comparative RNA-sequencing of mouse vHpc and human LCLs. **a** Heat map depicts the fold change of common genes with an uncorrected *p*-value < 0.05 found in mouse vHpc and human LCLs. Whole-genome sequencing of LCLs and its mouse orthologues were normalized by DESeq2 and matched to find differentially expressed genes in the following comparisons: (i) OVX Het-Met mice treated with vehicle versus OVX WT mice treated with vehicle, and PMDD LCLs treated with vehicle versus control LCLs treated with vehicle. (ii) OVX Het-Met mice treated with E2 versus OVX WT mice treated with E2, and PMDD LCLs treated with E2 versus control LCLs treated with E2. (iii) OVX WT mice treated with E2 versus OVX WT mice treated with vehicle, and control LCLs treated with E2 versus control LCLs treated with vehicle. (iv) OVX Het-Met mice treated with E2 versus OVX Het-Met mice treated with vehicle, and PMDD LCLs treated with E2 versus PMDD LCLs treated with vehicle. Red rectangles highlight genes with same directionality. 548 common genes were found across the four comparisons regardless of fold change directionality. **b** Pie-chart depicting the enrichment analysis of the top 10 pathways from Database for Annotation, Visualization and Integrated Discovery (DAVID) revealed epigenetic pathways that were common to mouse and human. **c** Venn diagram depicting the number of genes altered in E2-treated PMDD subjects (pink circle), E2-treated OVX Het-Met mice (red circle), and in both species (pink-red overlap) (uncorrected *p*-value < 0.05) when compared to their respective treatment-matched controls. We found that E2 induced 3188 genes in PMDD women and 1385 genes in Het-Met mice, with 416 common to both species. **d** Scatter plots depicting a positive correlation of Mtf2 with emotionality *z*-scores for Het-Met but not WT mice regardless of treatment. **e** GeneMania (genemania.org) was used to generate a gene network analysis inputting 9 epigenetic genes found in the human and mouse comparison. All network categories were assigned an equal weight, with the weighting also evenly distributed among networks within each category. Black circles with gray stripes indicate the input genes. Black circles with no stripes indicate additional genes identified in the network—a larger circle reflects a higher rank of connection with the input genes. A thicker connecting bar between nodes reflects a stronger relationship between those two nodes. The expanded network highlighted 18 common genes between mouse and humans. Circles highlighted in yellow represent ESC/E(Z) complex genes. E2 add-back induced intrinsic, common epigenetic modifiers in the vHpc of OVX Het-Met mice and LCLs of PMDD women

In a recent paper by Dubey et al. [18], the authors have found that genes of the estrogen-sensitive epigenetic ESC/E(Z) complex were expressed differentially at the RNA and protein levels in lymphoblastoid cell lines (LCLs) isolated from women with PMDD compared to those from non-affected control women. We used our BDNF Val66Met versus WT genotype RNA-seq data on hippocampus to determine a possible intersection with the behavioral and transcriptional traits of women with PMDD. When orthologues of OVX Het-Met mice and PMDD LCLs were compared to OVX WT mice and control LCLs, we identified 29 and 416 common genes under vehicle (Fig. 5a,i) and E2 (Fig. 5a,ii) treatment, respectively. Out of 445 genes, 185 genes expressed the same fold change directionality in human and mouse tissue. Mouse and human also displayed gene expression overlap after E2 add-back when compared to their respective untreated cohort (Fig. 5a,iii,iv). Specifically, we found that E2 add-back induced 23 common genes between OVX WT mice and control LCLs, and 80 common genes between OVX Het-Met mice and PMDD LCLs. Out of these 103 genes, 68 expressed the same fold change directionality in human and mouse tissue.

A GO analysis of commonly regulated genes revealed, among the top 10 enriched pathways, two major epigenetic clusters, namely, transcription and covalent chromatin modification (Fig. 5b). Based on our computational epigenetic predictions (Figs. 4a and 5a), we searched for common epigenetic modifiers in the human and mouse comparisons (Fig. 5c). E2 add-back induced 9 epigenetic genes (SMARCA4, CHD3, HDAC6, HDAC7, HDAC10, KDMC5, SETD1B, SETD7, and MTF2) that were common between OVX Het-Met mice and PMDD LCLs when compared to their respective E2-treated controls. Of note, Mtf2 is a core gene of the ESC/E(Z) complex. Curiously, Mtf2 levels in mice positively correlated with emotionality in Het-Met but not in WT mice regardless of treatment (Fig. 5d). These common epigenetic genes were entered into GeneMANIA (genemania.org), a software that computes connection density and strength of the input genes and identifies genes that are strongly correlated to the interaction network. The epigenetic interactome that overlapped human and mouse included MEF2C, HDAC1, USP47, PPARD, ARID1A, HDAC3, SMARCB1, PRKD2, SMARCC2, NCOR2, HDAC11, PLAA, SUPT6H, and PITRM1, as well as the ESC/E(Z) complex genes SUZ12, EZH2, HDAC2, and JARID2 (Fig. 5e). Thus, E2 add-back induced intrinsic, common epigenetic modifiers in the vHpc of OVX Het-Met mice and LCLs of PMDD women that transcended species and cell types.

## Discussion

We report that BDNF Het-Met female mice show a considerable degree of behavioral lability after non-invasive E2 add-back in OVX mice [29] when compared to OVX WT female mice. This mouse paradigm sheds light from an epigenetic perspective on PMDD in humans, where ovarian suppression has been used to treat negative symptoms associated with PMDD, such as depressive mood, anxiety, irritability, and other somatic symptoms [16]. Women with PMDD display recurrence of negative symptoms when treated with E2 [16, 30]. Besides parallels in the lability of behavior under E2 in Het-Met female mice and women with PMDD, we also found parallels between expression of epigenetic regulatory genes in the vHpc of OVX WT and OVX Het-Met mice and in LCL cultures from healthy women and women with PMDD, respectively.

Physiologically, in Het-Met mice, the BDNF Met allele is responsible for weight gain regardless of E2 treatment, although we also confirmed that E2 prevented OVX-induced body weight gain regardless of genotype. Behaviorally, we report that OVX Het-Met mice showed increased lability of anxiety-like and depressive-like behaviors under E2 add-back, consistent with reports on intact mice. For example, it has been reported in mice that the BDNF Met allele interacts with the E2 surge/drop during the estrous cycle, leading to increased anxiety and impaired cognitive performance [5–7]. Indeed, homozygous Met mice display increased anxiety in the open field and elevated plus maze during estrus, when circulating levels of E2 decline from its peak [5]. In female BDNF Met mice, spatial memory performance varies depending on the stage of estrous cycle [7]. Curiously, intact Het-Met female mice display cognitive deficits that are corrected by ovariectomy alone [6]. Together with our report, these findings suggest that, similar to PMDD women, behavioral impairment in Met carriers arise from the presence of E2 [16, 30].

The BDNF Met genotype and ovarian steroids also interact to modulate working memory-related hippocampal function in humans [8]. A recent study examined the interaction between ovarian hormones and the Met allele in asymptomatic women. Opposite to controls (BDNF Val66Val), Met carriers displayed elevated PET activity in the hippocampus with E2 add-back during a working memory task. Curiously, this increase did not occur in the absence of ovarian hormones [8].

Because hippocampal regulation is critical for estrogen actions on mood and cognitive performance in BDNF Met carriers, we investigated the Het-Met transcriptome of the vHpc, a sub-region that is peculiarly involved in memory-related to stress and emotion [31]. OVX WT and OVX Het-Met mice displayed markedly distinct gene expression profiles both at baseline and after treatment with E2. Importantly, E2-induced unique gene sets in OVX Het-Met mice, and did not induce other genes that were selectively regulated in OVX WT mice. For example, E2-induced *Egr3* and *Egr4* in OVX WT mice but not in OVX Het-Met mice. Members of the *Egr* family are involved in brain plasticity and neuropsychiatric disorders related to sex hormones [32, 33]. Thus, it is likely that E2-related behavioral impairment is associated with a lack of plasticity in Het-Met mice in response to E2.

Among the other networks effected by E2 in a genotype-dependent manner, we found genes belonging to the glutamate/GABA network, such as *Gria2* and *Gabarap*, that were regulated by E2 in OVX WT mice but not affected by E2 in OVX Het-Met mice. Dysregulation of excitatory and inhibitory neurotransmission and their receptors underpins the phenotype of Met carriers [34, 35]. In Het-Met mice, the hippocampal expression of type 2 metabotropic glutamate receptor is epigenetically regulated via histone-3-lysine-27-acetylation after acute stress [35]. Thus, epigenetic plasticity in the hippocampus is integral to the behavioral and molecular phenotype of Het-Met mice.

Notably, we found that epigenetic modifiers were also expressed in a genotype-by-treatment manner: namely, genes affected by E2 in OVX WT mice but not in OVX Het-Met mice. Among those genes we identified *Nat8l* which has been recently associated with depression-like behavior in mice [36], and *Mecp2*, a gene tasked with methylation of estrogen receptor alpha [37]. Surprisingly, many genes were expressed similarly in E2-treated OVX WT and in OVX Het-Met mice in the absence of E2 (a “pre-estrogen state”; see below), suggesting that behavioral impairment observed during E2 add-back in OVX Het-Met mice may result from a lack of E2-related plasticity in Met carriers. Another cluster of epigenetic modifiers, the ESC/E(Z) complex genes, was differentially expressed in OVX Het-Met mice. Interestingly, using LCLs isolated from women with PMDD, Dubey et al. [18] demonstrated that women with PMDD manifest a cellular difference in the ESC/E(Z) complex expression.

Although cellular models have limitations for the study of brain disorders, it is noteworthy that the epigenetic signature of OVX Het-Met mice intersected with that of PMDD LCLs in a cross-species and cross-tissues comparison. For example, the histone-binding MTF2 was upregulated under E2 in both OVX Het-Met mice and PMDD LCLs when compared to their respective E2-treated OVX WT mice and control LCLs. We found that, in mice, an increased expression of MTF2 was associated with increased emotionality in Het-Met but not in WT mice. This warrants further investigation as to whether higher levels of Mtf2 are associated with increased risk of mood disorders in humans. MTF2 stimulates the trimethylation activity of EZH2 toward the H3K27me2 substrate [38, 39]. Histone methyltransferase activity of the ESC/E(Z) complex also includes EZH1, EED, and SUZ12, and involves the chromatin-binding regulator JARID2 [40, 41]. Interestingly, we identified EZH1, EZH2, EED, SUZ12, and JARID2 in the expanded epigenetic interactome of E2-treated OVX Het-Met mice and of PMDD LCLs treated with E2. This network also comprised the SET-domain methyltransferases SETD7, SETD1B, and SETD1A, suggesting that increased steroid sensitivity in Het-Met mice and women with PMDD is potentially associated with similar cross-tissues and cross-species methyltransferase activity.

Another group of epigenetic modifiers encompassed the SMARCA cluster, a chromatin-remodeling complex that is regulated by steroid hormones [42–44]. SMARCA genes are also required for antidepressant and antipsychotic activity [45, 46]. Oh et al. [46] demonstrated that SSRI-induced neurogenesis and behavioral responses are abolished by constitutive knockout of *SMARCA3*, while antipsychotics increase expression of *Smarca2* in the mouse brain [45].

Regarding the cross-species and cross-tissues comparison, there was a higher number of genes with the same directionality induced by E2 in OVX Het-Met mice and PMDD LCLs (76%) compared to the number of genes induced by E2 in WT and control LCLs (30%). The highest similarity in gene expression occurring during E2 parallels the estrogen-related behavioral reactivity observed in OVX Het-Met mice and women suffering from PMDD.

Finally, we observed that the majority of the genes incorporated in the ESC/E(Z) complex were regulated by E2 in OVX WT mice and also expressed in OVX Het-Met mice treated with vehicle, i.e., genes that were induced by E2 in OVX WT mice were also differentially expressed in OVX Het-Met mice at baseline. This “pre-estrogen state” is reminiscent of the “pre-stressed” phenotype observed in Het-Met mice, in which the same genes that are expressed without applied stress in Het-Met mice are also induced in WT mice by an acute stressor [6]. Because E2 is reported to compete with corticosterone to inhibit glucocorticoid signaling [34], it is possible that the pre-stressed state and behavioral alteration of Het-Met mice result from the disruption of E2 signaling in the hippocampus. This hypothesis warrants further investigation to discover alternative interpretations as to why the epigenetic signature of Het-Met mice intersects that of LCLs from women with PMDD. That is, PMDD parallels a “pre-stressed state” [6], which, in turn induces higher risk for mood disorders and cognitive dysfunctions mostly associated with the luteal phase of menstruation.

Although PMDD has features of a “pre-stress” or “pre-estrogen” state, it does not necessarily indicate that PMDD is a direct result of the BDNF Met allele. The BDNF genotype has not been mapped in every PMDD subject or control, but, in the subjects that were genotyped, BDNF Met was equally distributed across controls and women with PMDD (2 Het-Met women in either group, see Supplementary Material and Methods). This distribution indicates that: (i) it is unlikely that the BDNF Met genotype drives the epigenetic intersection between mice and women; (ii) the number of Het-Met women is statistically insufficient to generate a subset genotypic analysis. Further studies of genotype are needed, since the BDNF Met allele is a risk factor for several other neuropsychiatric disorders, including depression, schizophrenia, Parkinson, and Alzheimer’s disease [47–49]. Yet, at the epigenetic level, our findings generate testable hypotheses concerning conserved, cross-species and cross-tissues epigenetic factors underlying behavioral sensitivity to ovarian hormones and may open a window to novel targets for therapy.

## Electronic supplementary material

Tables 1-3

Supplementary Material and Methods

Supplementary Figures
