# Supplementary material for: Epigenetic intersection of BDNF Val66Met genotype with premenstrual dysphoric disorder transcriptome in a cross-species model of estradiol add-back
Source: Mol Psychiatry. 2018 Oct 24;25(3):572–83. doi: 10.1038/s41380-018-0274-3 (PMC7042769; doi:10.1038/s41380-018-0274-3)
Supplement: Supplementary file 1 — Tables 1-3 [file 41380_2018_274_MOESM1_ESM.docx]

**Supplementary Table 1.** **Light-dark box test**

| **Group** | **Time in light box (%)** | **Latency to enter the light box (s)** |
| --- | --- | --- |
| WT+Veh | 23.57 ± 1.93 | 46.37 ± 8.67 |
| WT+E2 | 24.89 ± 3.70 | 41.12 ± 6.38 |
| Het-Met+Veh | 28.76 ± 5.34 | 44.68 ± 7.83 |
| Het-Met+E2 | 26.98 ± 2.81 | 45.21 ± 11.27 |

The table displays the % of time spent in the light box and the latency to enter the light box. WT: OVX WT mice. Het-Met: OVX Het-Met mice. Veh: Vehicle. E2: Estradiol.

**Supplementary Table 2.** **Overlapping genes induced by E2 within genotype**

| **Gene** | **WT** | **Het-Met** |
| --- | --- | --- |
|  | **Up** | |
| *Ecel1* | 2.33 | 1.81 |
| *Sfmbt2* | 1.79 | 1.47 |
| *LOC100861635* | 1.49 | 1.41 |
| *LOC101056185* | 1.58 | 1.46 |
| *LOC100861715* | 1.49 | 1.42 |
| *Scn5a* | 2.18 | 1.32 |
| *LOC101055684* | 1.44 | 1.48 |
| *Pqlc3* | 1.58 | 1.32 |
| *Cartpt* | 1.64 | 1.58 |
| *Gm7120* | 1.48 | 1.52 |
| *LOC101055764* | 1.51 | 1.57 |
| *Ccl28* | 1.40 | 1.34 |
| *Erdr1* | 1.42 | 1.37 |
|  | **Down** | |
| *Myoc* | 1.58 | 1.31 |

Genes that are upregulated (Up) or downregulated (down) by E2 in both genotypes were obtained by performing a fold-change analysis using Strand NGS (Z-score<0.05, absolute fold change>1.3). WT: OVX WT mice treated with E2 vs OVX WT mice treated with vehicle. Met: OVX Het-Met mice treated with E2 vs OVX Het-Met mice treated with vehicle.

**Supplementary Table 3**. **Overlapping genes between OVX WT and OVX Het-Met mice under vehicle or E2**

| **Gene** | **Vehicle** | **Estradiol** |
| --- | --- | --- |
|  | **Up** | |
| *Gm5415* | 2.49 | 2.18 |
| *Myoc* | 1.42 | 1.70 |
| *Dnajc17* | 1.40 | 1.33 |
| *Pla2g4e* | 2.29 | 1.79 |
| *Gbp3* | 1.45 | 1.42 |
| *Gabra2* | 1.33 | 1.48 |
| *Asprv1* | 1.51 | 1.46 |
| *Ntf3* | 1.86 | 1.36 |
| *B3gnt9-ps* | 1.37 | 1.33 |
| *Cyba* | 1.38 | 1.31 |
| *Snhg10* | 1.85 | 1.40 |
| *Psme2* | 1.38 | 1.41 |
| *Rec8* | 1.44 | 1.37 |
| *Gm4285* | 1.31 | 1.35 |
| *Ccdc122* | 1.56 | 1.59 |
| *Rpl30* | 1.36 | 1.63 |
| *D330041H03Rik* | 4.71 | 7.03 |
| *Aif1* | 1.40 | 1.35 |
| *A330093E20Rik* | 1.31 | 1.53 |
|  | **Down** | |
| *Xkr4* | 1.41 | 1.54 |
| *Rims1* | 1.35 | 1.36 |
| *Bmpr2* | 1.54 | 1.81 |
| *Nbeal1* | 1.35 | 1.40 |
| *Klf7* | 1.52 | 1.42 |
| *Erbb4* | 1.41 | 2.01 |
| *Hdac4* | 1.38 | 1.45 |
| *Cntnap5b* | 1.44 | 1.32 |
| *Cntnap5a* | 1.40 | 1.50 |
| *Syt2* | 1.41 | 1.40 |
| *Tnr* | 1.49 | 1.72 |
| *Zbtb37* | 1.31 | 1.65 |
| *Kcnh7* | 1.55 | 1.64 |
| *Fign* | 1.55 | 1.72 |
| *2810002D19Rik* | 1.45 | 1.37 |
| *Ccdc73* | 1.51 | 1.42 |
| *1700020I14Rik* | 1.43 | 1.40 |
| *Ptprt* | 1.37 | 1.70 |
| *Gm16119* | 1.49 | 1.42 |
| *C030034L19Rik* | 1.41 | 1.35 |
| *Gatad2b* | 1.37 | 1.51 |
| *Wnt2b* | 1.55 | 1.30 |
| *Kcna3* | 1.66 | 1.87 |
| *Gm4392* | 1.49 | 1.64 |
| *Dnajb14* | 1.41 | 1.73 |
| *Lrrc7* | 1.36 | 1.31 |
| *Nkain3* | 1.33 | 1.74 |
| *LOC100861635* | 1.55 | 1.64 |
| *LOC101056185* | 1.77 | 1.92 |
| *Nr4a3* | 1.35 | 1.67 |
| *Tmem245* | 1.41 | 1.67 |
| *6430704M03Rik* | 1.31 | 1.34 |
| *Eif2c3* | 1.48 | 1.40 |
| *Csmd2* | 1.63 | 1.37 |
| *Gm3414* | 1.60 | 1.37 |
| *Ksr2* | 1.51 | 1.52 |
| *Hipk2* | 1.54 | 1.58 |
| *Gm5567* | 1.39 | 1.72 |
| *Grid2* | 1.56 | 1.46 |
| *Cacna1c* | 1.36 | 1.31 |
| *Grin2b* | 1.60 | 2.20 |
| *Bicd1* | 1.36 | 1.33 |
| *D630041G03Rik* | 1.92 | 1.50 |
| *Zfp551* | 1.30 | 1.32 |
| *Tenm4* | 1.31 | 1.31 |
| *Srcap* | 1.33 | 1.31 |
| *Gpr26* | 1.48 | 1.66 |
| *D130040H23Rik* | 1.74 | 1.74 |
| *Sntb2* | 1.38 | 1.30 |
| *Gm1943* | 1.59 | 1.62 |
| *Gan* | 1.65 | 1.73 |
| *Igsf9b* | 2.07 | 1.42 |
| *Cbl* | 1.37 | 1.59 |
| *Rora* | 1.34 | 1.77 |
| *Zfp167* | 1.39 | 1.37 |
| *D10Bwg1379e* | 1.51 | 1.47 |
| *Slc16a7* | 1.36 | 1.36 |
| *Klhl11* | 1.34 | 1.54 |
| *Helz* | 1.36 | 1.58 |
| *Gdap10* | 1.78 | 1.39 |
| *Trip11* | 1.39 | 1.39 |
| *Mrs2* | 1.37 | 1.36 |
| *Shc3* | 1.39 | 1.58 |
| *Zfp369* | 1.32 | 1.37 |
| *Gm7120* | 1.90 | 1.84 |
| *LOC101055764* | 1.86 | 1.79 |
| *Ccl28* | 2.10 | 2.19 |
| *Kcnma1* | 1.35 | 1.40 |
| *Xpo4* | 1.38 | 1.50 |
| *Sacs* | 1.53 | 1.56 |
| *Adra1a* | 1.41 | 1.72 |
| *Dgkh* | 1.56 | 1.39 |
| *Klf12* | 1.46 | 1.50 |
| *Hs6st3* | 1.54 | 1.55 |
| *Plcxd3* | 1.50 | 1.63 |
| *Lmbrd2* | 1.44 | 1.52 |
| *Cdh6* | 1.90 | 1.43 |
| *Cdh12* | 1.34 | 1.44 |
| *Samd12* | 1.51 | 1.37 |
| *Kcnq3* | 1.48 | 1.58 |
| *Fam135b* | 1.61 | 1.38 |
| *Kcnk9* | 1.39 | 1.40 |
| *Eif2c2* | 1.34 | 1.46 |
| *Ttll8* | 1.86 | 1.36 |
| *Grin2a* | 1.54 | 1.66 |
| *Heg1* | 1.40 | 1.35 |
| *Zbtb20* | 1.55 | 1.98 |
| *Zdhhc23* | 1.45 | 1.40 |
| *Epha6* | 1.34 | 1.50 |
| *Nrip1* | 1.38 | 1.50 |
| *Ncam2* | 1.32 | 1.31 |
| *Lnpep* | 1.60 | 1.58 |
| *H2-T24* | 1.61 | 1.58 |
| *St6gal2* | 1.47 | 1.43 |
| *Plin4* | 1.39 | 1.99 |
| *Cntnap5c* | 1.53 | 1.49 |
| *Gm10493* | 1.39 | 1.31 |
| *Egr1* | 1.47 | 1.46 |
| *Pcdhb2* | 1.47 | 1.76 |
| *Kctd16* | 1.39 | 1.39 |
| *Dpysl3* | 1.33 | 1.35 |
| *Dok6* | 1.33 | 1.81 |
| *Npas4* | 1.34 | 1.49 |
| *Ptar1* | 1.41 | 1.78 |
| *Gm340* | 1.35 | 1.40 |
| *Lancl3* | 1.45 | 1.45 |
| *Slc9a7* | 1.36 | 1.59 |
| *Cdr1* | 1.48 | 1.31 |
| *Aff2* | 1.53 | 1.51 |
| *Klhl15* | 1.40 | 1.65 |
| *Zc3h12b* | 1.42 | 1.34 |
| *Cdkl5* | 1.69 | 1.87 |
| *Erdr1* | 1.33 | 1.37 |

Genes that are upregulated (Up) or downregulated (Down) in Het-Met mice within treatment were obtained by performing a fold-change analysis using Strand NGS (Z-score<0.05, absolute fold change>1.3). Vehicle: OVX Het-Met mice treated with vehicle vs OVX WT mice treated with vehicle. E2: OVX Het-Met mice treated with E2 vs OVX WT mice treated with E2.
