# Supplementary material for: Epigenetic intersection of BDNF Val66Met genotype with premenstrual dysphoric disorder transcriptome in a cross-species model of estradiol add-back
Source: Mol Psychiatry. 2018 Oct 24;25(3):572–83. doi: 10.1038/s41380-018-0274-3 (PMC7042769; doi:10.1038/s41380-018-0274-3)
Supplement: Supplementary file 2 — Supplementary Material and Methods [file 41380_2018_274_MOESM2_ESM.pdf]

SUPPLEMENTARY MATERIAL

Surgery and Treatment

To control for litter-specific effects[1, 2], mice in each group were selected from across multiple litters. Animals were group housed (n=4–5) in standard cages (28.5x17x13cm) and were kept on a 12-h light-dark cycle (lights off 7:00pm) in a temperature-controlled room maintained at 21±2°C. Food and water were available *ad libitum*. Seventy to eighty days-old female mice were anesthetized with a solution of ketamine (75 mg kg^{-1}) and xylazine (7.5 mg kg^{-1}), hair was removed from each flank using an electric shaver, and the ovaries were removed via a small incision made on each flank. Mice were allowed to recover for 2 weeks before E2 treatment began. WT and Het-Met mice were treated with either 300 nM β-E2 in 0.1% ethanol (0.7 μg/day), or with 0.1% ethanol (vehicle) solution, administered *ad libitum* in cage water bottles. It has been reported that this dose leads to physiological uterine weight and E2 levels similar to sham-operated treated mice[3]. Solutions were changed twice weekly, and treatment continued for 6 weeks. Mice were weighed weekly starting from the first day of treatment. Body weight and uterine weight were measured to validate E2 add-back (Supplementary Fig. 1A, B). Behavioral assessment began during the third week of treatment. Based on previous behavioral studies, we found that a sample size of 8 to 12 mice per group allows us to reliably detect changes of the magnitude we are examining (α=0.05). Variance is similar among the groups that are being compared. All procedures were performed in accordance with the National Guidelines on the Care and Use of Animals and a protocol approved by The Rockefeller University Animal Care and Use Committee.

Light-Dark Box Test

Mice were tested in an arena with an open white-wall light box and a covered black-wall dark box (l=29cm, w=29cm) for 5 minutes and videotaped with a camera fixed on the ceiling above the arena (50±10 lux). % Time spent in the light box, latency to enter the dark box, and latency to reenter the light box were recorded and scored using Ethovision software.

Open Field Test

Mice were placed into an empty arena (l=45cm, w=45cm) and allowed to explore for 10 minutes (50±10 lux). The test was recorded using a video camera placed over the cage. The time spent in the center of the arena, the total distance moved, the number of center visits and the time spent in the corner were analyzed using Ethovision software.

Splash Test

The splash test was performed as described by Isingrini *et al.* (2010)[4] with minor changes. Mice were habituated for 30 minutes to a new, empty testing cage. Mice were then sprayed on the hindquarters with a 10% (w/v in water) sucrose solution, and immediately placed in the testing cage for 5 minutes (50±10 lux). Grooming behavior was recorded with a video camera placed over the cage. The latency to the first grooming session, the total time spent grooming, and the number of grooming sessions were scored by an experimenter blind to the experimental groups and conditions.

Selection of participants for generation of lymphoblastoid cell lines (LCLs)

Women between the ages of 18 and 48 years who were medication-free (at time of recruitment), with regular menstrual cycles (range 21 to 35 days), not medically ill and not pregnant were included in the clinical study[5]. Women with PMDD were self-referred in response to newspaper advertisements or were referred by their physician. The diagnosis of PMDD was confirmed prospectively prior to entry into this study. Women without PMDD (referred to as Control women) were recruited, and studied, in parallel (for details of selection criteria see Dubey *et al.*, 2017[6]). Lymphoblastoid cell lines (LCLs) were created in participants who completed participation in the GnRH agonist-induced hormone manipulation protocol[5]: women with PMDD (n=16) were selected based on suppression of PMDD symptoms during leuprolide treatment and recurrence of symptoms during the E2 add-back condition, and women without PMDD (Controls [n=12]) were selected based on the absence of symptoms throughout the entire protocol[5]. All blood samples used to create LCLs were collected once for each woman after completion of the hormone stimulation studies which both cases and controls underwent. Dubey *et al.* (2017)[6] employed in-house generated LCLs from patients and controls and cells were confirmed to be active and free-floating, but LCLs were not tested for mycoplasma nor authenticated. Under this protocol, rate of successful LCL transformation approaches 100%. The estradiol exposure therefore was to the LCLs only. The BDNF genotype was not mapped in all PMDD or control subjects in this study. Amongst the 12 control women, of which 7 LCLs were untreated and 5 LCLs were treated with E2, one woman was not genotyped while two women had the BDNF Met allele. In the PMDD groups, 8 LCLs were untreated and 8 LCLs were treated with E2. In women with PMDD, two women were not genotyped and two women carried the BDNF Met allele. All women received remuneration according to guidelines from the NIH Healthy Volunteer Office. The study protocol was reviewed and approved by the National Institute of Mental Health Institutional Review Board, and all women gave written informed consent. For a detailed description of the experimental protocol see Dubey *et al.* (2017)[6]. NIMH Protocols NCT00001259 and NCT00001322.

Data availability

The RNA-sequencing data discussed in this publication have been deposited in NCBI's Gene Expression Omnibus and are accessible through GEO Series accession number GSE121412. (https://www.ncbi.nlm.nih.gov/geo/query/acc.cgi?acc=GSE121412). All other relevant data are available from the authors upon reasonable request.

1. Becker G, Kowall M. Crucial role of the postnatal maternal environment in the expression of prenatal stress effects in the male rats. *Journal of comparative and physiological psychology* 1977; **91**(6)**:** 1432-1446.

2. Chapman RH, Stern JM. Failure of severe maternal stress or ACTH during pregnancy to affect emotionality of male rat offspring: implications of litter effects for prenatal studies. *Developmental psychobiology* 1979; **12**(3)**:** 255-267.

3. Levin-Allerhand JA, Sokol K, Smith JD. Safe and effective method for chronic 17beta-estradiol administration to mice. *Contemporary topics in laboratory animal science* 2003; **42**(6)**:** 33-35.

4. Isingrini E, Camus V, Le Guisquet AM, Pingaud M, Devers S, Belzung C. Association between repeated unpredictable chronic mild stress (UCMS) procedures with a high fat diet: a model of fluoxetine resistance in mice. *PloS one* 2010; **5**(4)**:** e10404.

5. Schmidt PJ, Nieman LK, Danaceau MA, Adams LF, Rubinow DR. Differential behavioral effects of gonadal steroids in women with and in those without premenstrual syndrome. *The New England journal of medicine* 1998; **338**(4)**:** 209-216.

6. Dubey N, Hoffman JF, Schuebel K, Yuan Q, Martinez PE, Nieman LK *et al.* The ESC/E(Z) complex, an effector of response to ovarian steroids, manifests an intrinsic difference in cells from women with premenstrual dysphoric disorder. *Molecular psychiatry* 2017; **22**(8)**:** 1172-1184.
