# Supplementary material for: Epigenetic intersection of BDNF Val66Met genotype with premenstrual dysphoric disorder transcriptome in a cross-species model of estradiol add-back
Source: Mol Psychiatry. 2018 Oct 24;25(3):572–83. doi: 10.1038/s41380-018-0274-3 (PMC7042769; doi:10.1038/s41380-018-0274-3)
Supplement: Supplementary file 3 — Supplementary Figures [file 41380_2018_274_MOESM3_ESM.pdf]

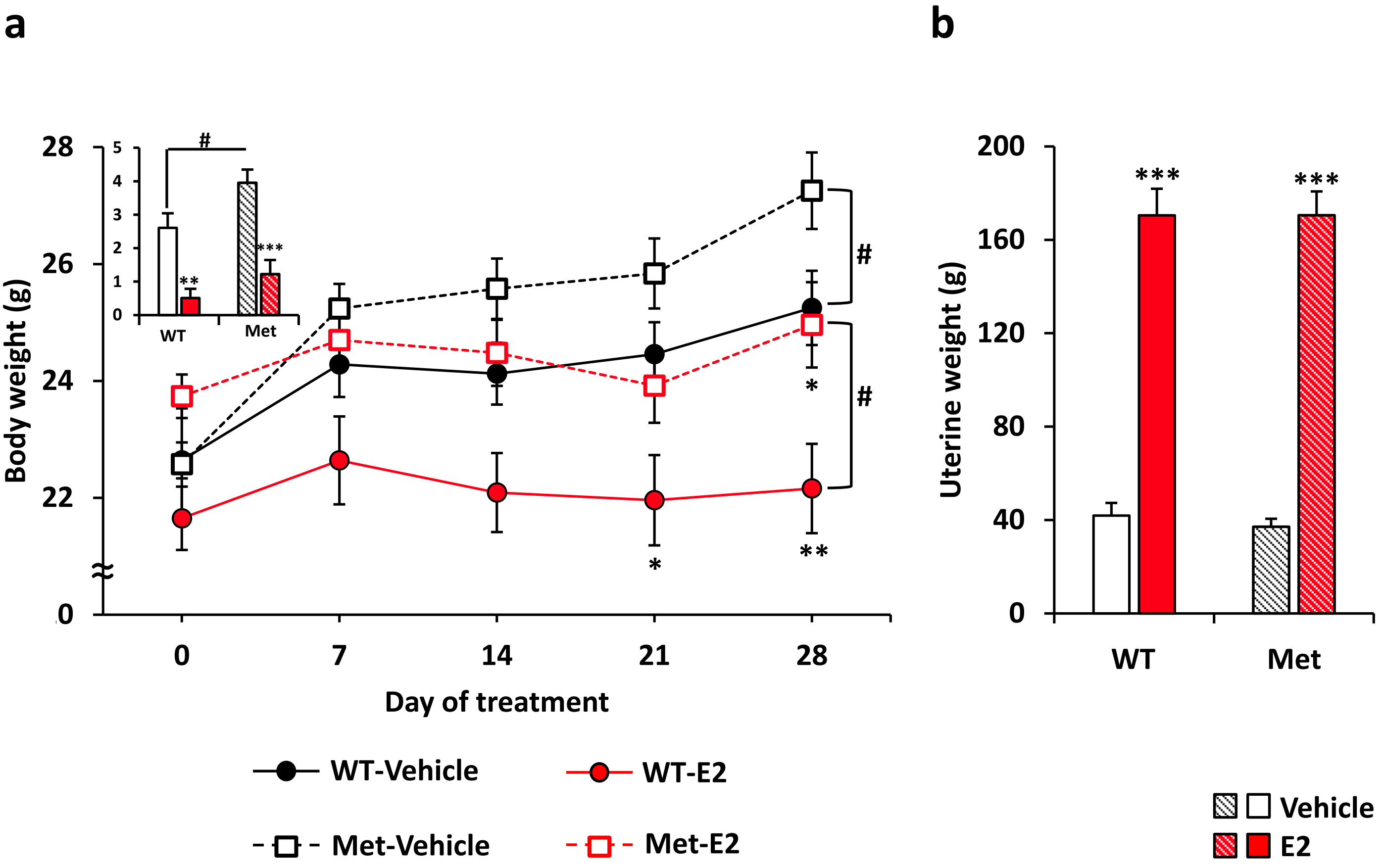

Supplementary Figure 1. Validation of E2 add-back protocol a) Body weight was measured once per week from day 0 till day 28 of treatment. E2 add-back prevented body weight increase in both OVX WT mice and OVX Het-Met mice. However, OVX Het-Met mice displayed higher body weight when compared to their matched-treated OVX WT mice (2-way ANOVA with repeated measures, $F_{(3,41)}$=6.26, p<0.01). Measurement of delta weight gain also indicated that E2 add-back prevented body weight increase over the 28 days of treatment in both groups (2-way ANOVA, treatment: $F_{(1,41)}$=37.02, *p*<0.001). Change in body weight in vehicle-treated OVX Het-Met mice was higher than body weight gain in vehicle-treated OVX WT mice (2-way ANOVA, genotype: $F_{(1,41)}$=6.72, *p*=0.01). b) E2-treated OVX mice displayed substantially higher uteri weight than their genotype-matched, vehicle-treated mice (2-way ANOVA, treatment: $F_{(1,39)}$=278.70, *p*<0.001). *p<0.05, **p<0.01, ***p<0.001 vs matched vehicle-treated mice. #p<0.05 vs matched-treated WT mice. WT: OVX WT mice. Met: OVX Het-Met mice.

Supplementary Fig.1

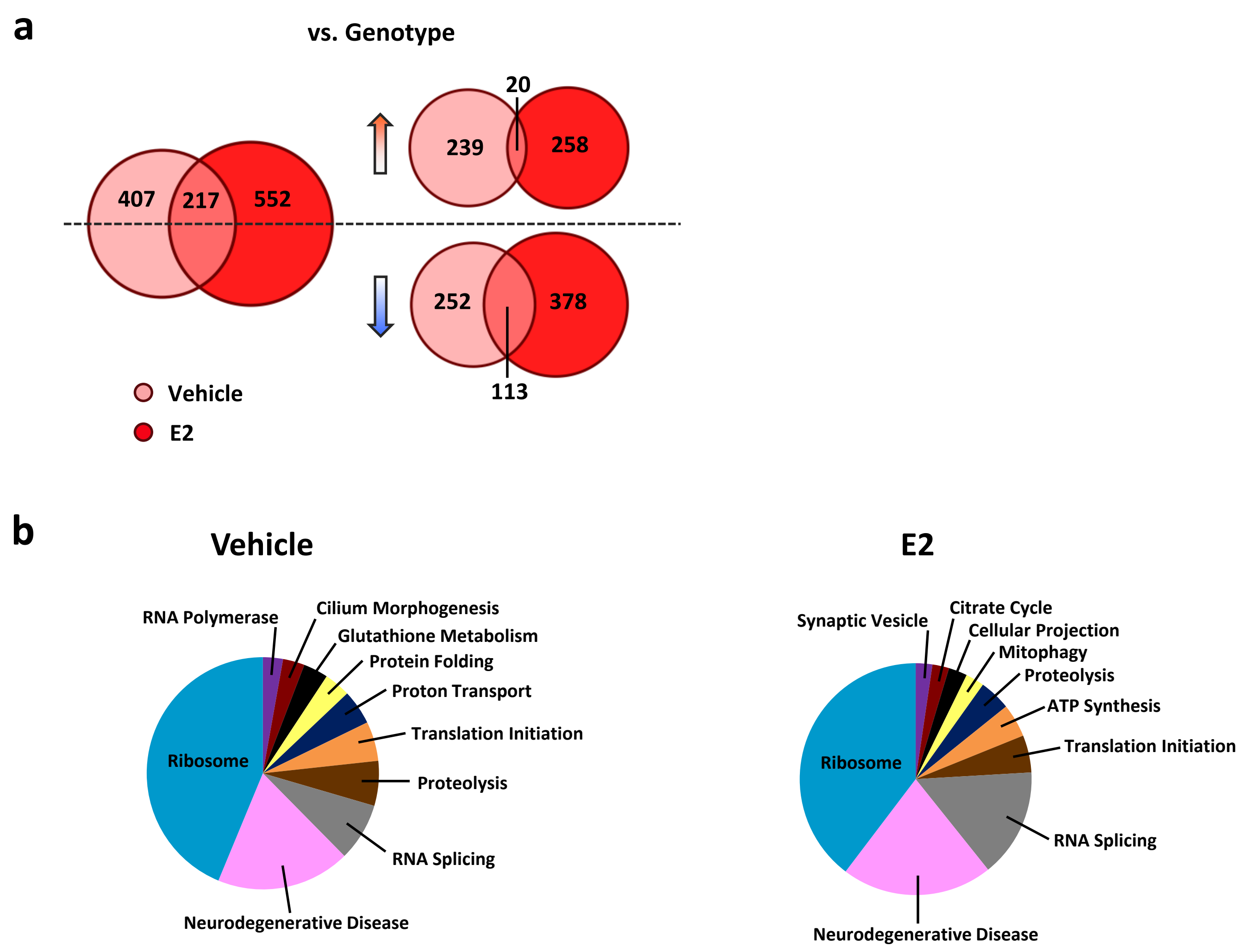

Supplementary Figure 2. Hippocampal whole genome sequencing in E2-treated OVX mice. a) Venn diagram depicting the number of genes expressed in vehicle-treated OVX Het-Met mice (light pink circle), expressed after E2-treatment in OVX Het-Met mice (red circle), and in both treatments (pink overlap) (Z-score < 0.05; absolute fold change > 1.3) when compared with treatment-matched OVX WT mice. There was a large discrepancy in the genes expressed under vehicle or E2 treatment. Indeed, 407 genes were differentially expressed in vehicle-treated Het-Met compared to WT mice and 552 genes were differentially expressed in E2-treated Het-Met compared to WT mice with 217 genes expressed differentially in Het-Met compared to WT mice independently of E2 add-back. However, when common treatment-regulated genes were separated based on the direction of their fold change, we found that 239 genes were upregulated in vehicle-treated Het-Met compared to WT mice and 258 genes were upregulated in E2 treated Het-Met compared to WT mice with 20 genes upregulated in Het-Met compared to WT mice independently of E2 add-back. Also, 252 genes were are downregulated in vehicle-treated Het-Met compared to WT mice and 378 were downregulated in E2-treated Het-Met compared to WT mice with 113 genes downregulated in Het-Met compared to WT mice independently of E2 add-back. This demonstrates that OVX WT and OVX Het-Met mice express distinct hippocampal genes regardless of circulating exogenous E2. b) Pie-charts depicting the enrichment analysis of the top 10 pathways from Database for Annotation, Visualization and Integrated Discovery (DAVID) revealed unique gene networks implicated in neuronal functions, cell signaling and glutamate GABA balance. Pathways were obtained by analyzing the genes in (clockwise): light pink circle in Fig (a) and red circle in Fig. (a). The Met allele induced similar pathways regardless of E2 add-back, such as ribosome, neurodegenerative disease, RNA splicing, translation initiation, and proteolysis. This indicates that genotype had a greater effect than treatment on gene expression.

Supplementary Fig. 2

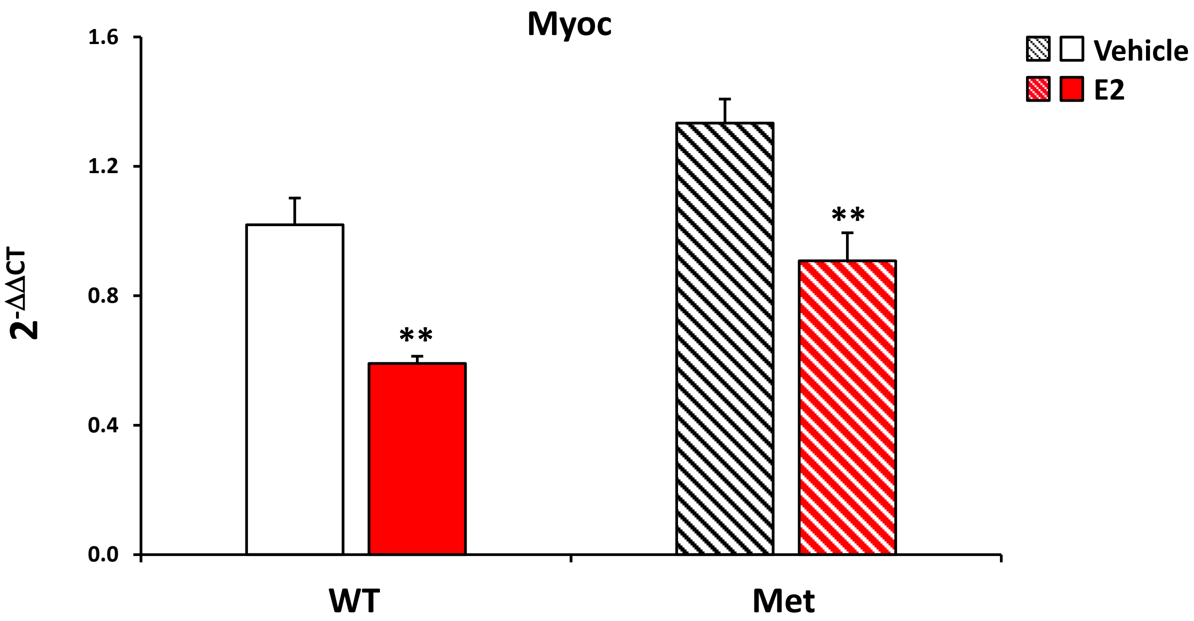

Supplementary Figure 3. qRT-PCR of *Myoc* depicting the fold change ($2^{-\Delta\Delta CT}$) against vehicle-treated OVX WT mice. This was used to validate RNA-sequencing. Myoc was present across the 4 comparisons and was among the top 100 most differentially expressed genes. E2 induces a downregulation of *Myoc* in either genotype as also predicted by RNA-sequencing. A two-way ANOVA followed by Newman-Keuls *post hoc* was used to establish statistical significance (treatment: $F_{(1,17)}$=31.46, p<0.0001). $\mathbf{2^{-\Delta\Delta CT}}$ = Fold Change vs. vehicle-treated OVX WT mice based on the delta of the threshold cycles. **p<0.01 vs matched vehicle-treated mice. WT: OVX WT mice. Met: OVX Het-Met mice.

Supplementary Fig. 3
